# Accuracy of Ultrasonography and Magnetic Resonance Imaging in the Diagnosis of Placenta Accreta

**DOI:** 10.1371/journal.pone.0094866

**Published:** 2014-04-14

**Authors:** Anne-Sophie Riteau, Mikael Tassin, Guillemette Chambon, Claudine Le Vaillant, Jocelyne de Laveaucoupet, Marie-Pierre Quéré, Madeleine Joubert, Sophie Prevot, Henri-Jean Philippe, Alexandra Benachi

**Affiliations:** 1 Department of Obstetrics and Gynecology, Hôpital Antoine Béclère, APHP, Clamart, France; 2 Department of Obstetrics and Gynecology, Hôpital Mère Enfant, Centre Hospitalier Universitaire, Nantes, France; 3 Department of Radiology, Hôpital Antoine Béclère, APHP, Clamart, France; 4 Department of Radiology, Hôpital Mère Enfant, Centre Hospitalier Universitaire, Nantes, France; 5 Department of Pathology, Hôpital Mère Enfant, Centre Hospitalier Universitaire Nantes, France; 6 Department of Pathology, Hôpital Antoine Béclère, APHP, Clamart, France; The Chinese University of Hong Kong, Hong Kong

## Abstract

**Purpose:**

To evaluate the accuracy of ultrasonography and magnetic resonance imaging (MRI) in the diagnosis of placenta accreta and to define the most relevant specific ultrasound and MRI features that may predict placental invasion.

**Material and Methods:**

This study was approved by the institutional review board of the French College of Obstetricians and Gynecologists. We retrospectively reviewed the medical records of all patients referred for suspected placenta accreta to two university hospitals from 01/2001 to 05/2012. Our study population included 42 pregnant women who had been investigated by both ultrasonography and MRI. Ultrasound images and MRI were blindly reassessed for each case by 2 raters in order to score features that predict abnormal placental invasion.

**Results:**

Sensitivity in the diagnosis of placenta accreta was 100% with ultrasound and 76.9% for MRI (P = 0.03). Specificity was 37.5% with ultrasonography and 50% for MRI (P = 0.6). The features of greatest sensitivity on ultrasonography were intraplacental lacunae and loss of the normal retroplacental clear space. Increased vascularization in the uterine serosa-bladder wall interface and vascularization perpendicular to the uterine wall had the best positive predictive value (92%). At MRI, uterine bulging had the best positive predictive value (85%) and its combination with the presence of dark intraplacental bands on T2-weighted images improved the predictive value to 90%.

**Conclusion:**

Ultrasound imaging is the mainstay of screening for placenta accreta. MRI appears to be complementary to ultrasonography, especially when there are few ultrasound signs.

## Introduction

Placenta accreta is a significant cause of maternal morbidity and mortality and is presently the most common reason for emergency postpartum hysterectomy. It is an abnormal attachment of the placenta to the myometrium, and occurs when a defect of the decidua basalis allows the chorionic villi to invade the myometrium. Placenta accreta is classified on the basis of the depth of myometrial invasion. In placenta accreta vera, villi are attached to the myometrium but do not invade the muscle. In placenta increta, villi partially invade the myometrium. The most severe type is placenta percreta, in which villi penetrate through the entire myometrial thickness or beyond the serosa. Identified risk factors include surgery, placenta previa and previous cesarean section [1], [2].

Its prevalence has risen tenfold in the United States over the past 50 years due to the rising number of cesarean deliveries. Previous cesarean section increases the odds of having placenta accreta about 8.7-fold [3]. As the number of cesarean sections increases, so does the risk. Accurate prenatal identification allows optimal obstetric management, because timing and site of delivery, availability of blood products, and recruitment of a skilled anesthesia and surgical team can be organized in advance [4], [5]. Ultrasonography and magnetic resonance imaging (MRI) have been used for the diagnosis of placenta accreta, but the accuracy of these two imaging techniques remains uncertain and is dependent on the skills of the sonographer or radiologist.

**Figure 1:**
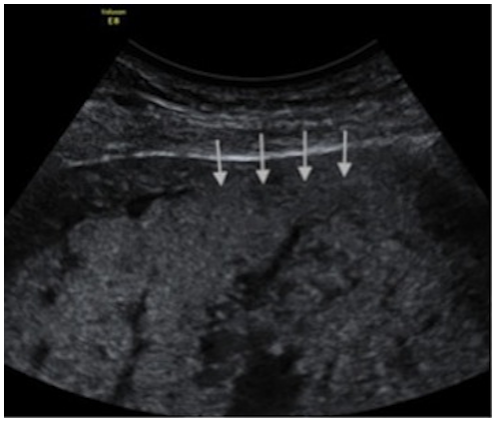
Loss of the normal retroplacental clear space on ultrasonography.

**Figure 2:**
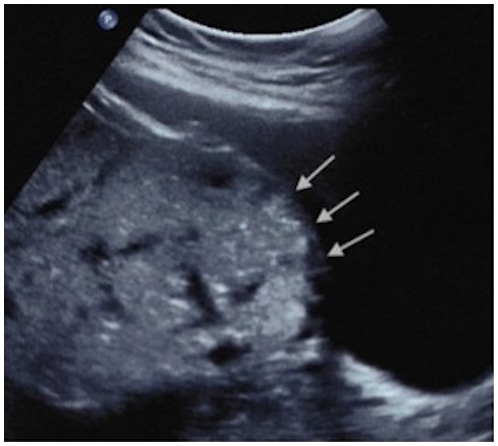
Uterine bulging and disruption of the hyperechogenic uterine serosa-bladder wall interface on ultrasonography.

**Figure 3:**
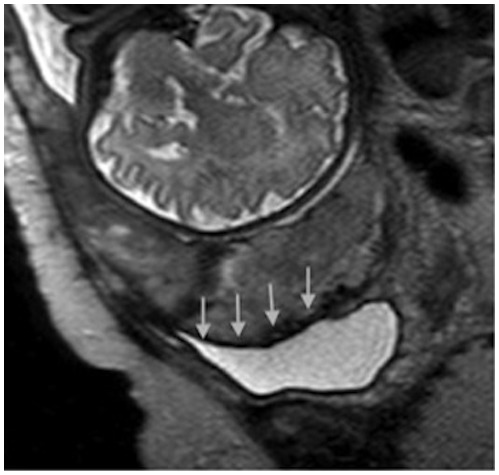
Uterine bulging into the bladder on MRI.

**Figure 4:**
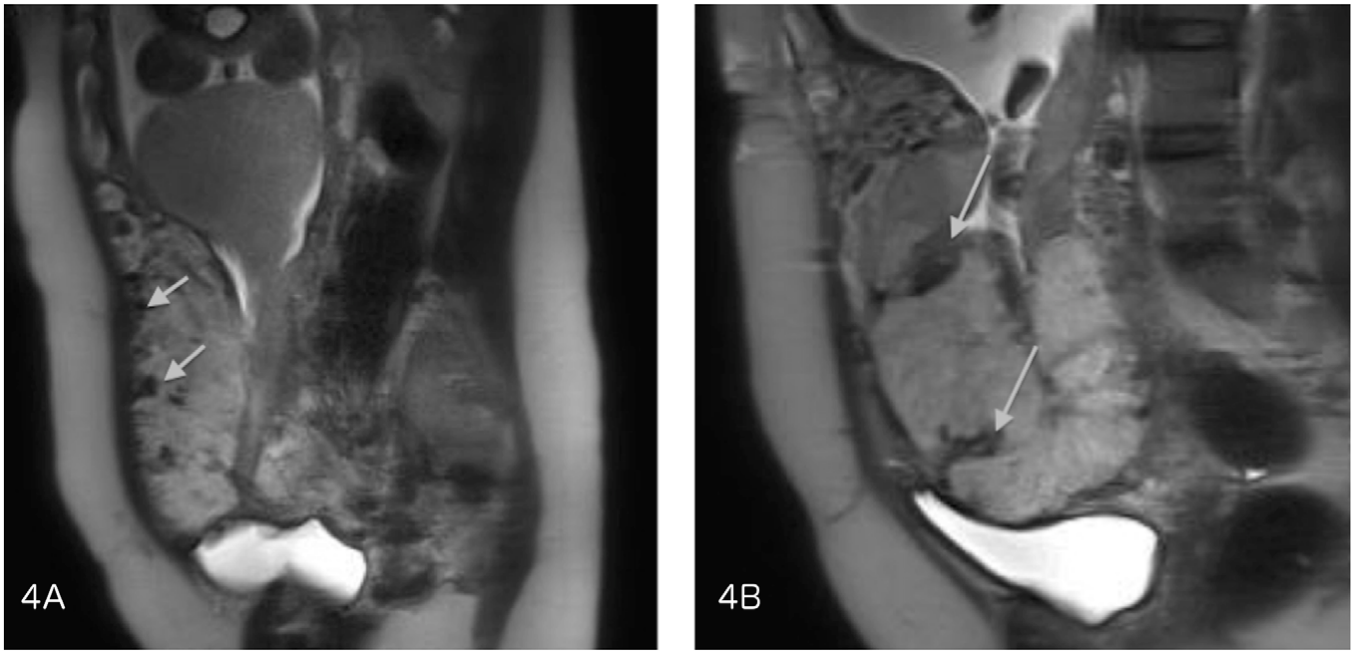
A–B - Dark intraplacental bands on T2-weighted images on MRI.

The sonographic characteristics of adherent placenta include: intraplacental lacunae, loss of the normal retroplacental clear space (Figure 1) and thinning or disruption of the hyperechogenic uterine serosa-bladder wall interface (Figure 2). Specific MRI findings in placenta accreta are: uterine bulging (Figure 3), heterogeneous signal intensity within the placenta and dark intraplacental bands on T2-weighted images (Figure 4 A–B).

The purpose of this study was to evaluate the accuracy of ultrasonography and MRI in the diagnosis of placenta accreta and to define the most relevant specific ultrasound and MRI features that may predict placental invasion.

## Material and Methods

We retrospectively reviewed the medical records of all patients referred for suspected placenta accreta to two university hospitals from January 2001 to May 2012. This study was approved by the institutional review board of the French College of Obstetricians and Gynecologists (Comité d’éthique de la recherche en gynécologie obstétrique [CEROG]), written informed consent was given by participants. Our study population included 42 pregnant women who had been investigated by both ultrasound and prenatal MRI. Medical chart review was used to record clinical information (Table 1).

**Table 1:** Clinical information.

	n = 42
**Average age (in years)**	34±4.7
**Gravidity**	4.2±2.3
**Parity**	2.1±1.5
**Previous cesarean delivery (%)**	37 (88%)
**Average gestational age at the time of diagnosis by ultrasonography (in weeks)**	28. 7
**Average gestational age at the time of MRI (in weeks)**	30.8
**Placental insertion (%)**	
Previa	32 (76.2%)
Anterior	26
Posterior	7
Low-lying	5 (11.9%)
Anterior	4
Posterior	2
Non-low-lying	5 (11.9%)
Anterior	4
Posterior	2
**Final diagnosis (%)**	
Placenta accreta/increta	16 (38%)
Placenta percreta	10 (24%)
Non-adherent placenta	16 (38%)
**Surgical management at delivery**	
Vaginal delivery	2
Conservative management	1
Hysterectomy	1
Cesarean delivery	40
Complete delivery	13
Incomplete delivery	3
Conservative management	14
Cesarean hysterectomy	10

Ultrasound and MRI were performed by obstetricians or radiologists experienced in abnormal adherent placenta. The equipment included the IU 22 system (Philips Medical Systems, Bothell, WA) and the GE Voluson 730 or E8 (GE Medical Systems, Zipf, Austria) with 4–9 MHz or 5–9 MHz transabdominal transducers, and 3–9 MHz and 4–8 MHz endovaginal transducers.

MRI was performed with a 1.5 Tesla scanner (Siemens Magnetom-Avanto, Siemens Magnetom-vision [Siemens Medical Solutions], Philips Achieva). The MRI protocols were similar in both hospitals and included T1-weighted sequences in the sagittal and axial planes, single-shot fast spin-echo T2-weighted MR sequences (HASTE, single shot TSE) and true fast imaging with steady-state precession (TrueFISP, FIESTA) in the axial, sagittal and coronal planes. 7 MRI scans were done after intravenous injection of gadolinium, 6 were MR diffusion-weighted imaging. No fetal sedation was used.

For the purpose of the study, ultrasound images and MRI were blindly reassessed by 2 raters with more than 5 years of experience in the evaluation of placentation disorders. They were blinded to the patient's diagnosis and were asked to score features previously described in the literature as useful for predicting placental invasion.

Placenta accreta was defined by clinical criteria at the time of delivery and by pathologic findings. The placenta was considered normal if it was easily removed during cesarean delivery without any bleeding complications. Ideally, the standard of reference for the diagnosis of abnormal adherent placenta is confirmation of the final histology after hysterectomy has been performed. However, hysterectomy is not always clinically indicated or possible and management should be conservative (decision to leave the placenta to involute in situ if bleeding is controlled). Therefore, in these cases pathologic examination was not available and the diagnosis was based on clinical information provided at the time of delivery and surgery. The placenta was considered as accreta when the delivery was impossible and as percreta when it was evident that the placenta had reached the uterine serosa or the adjacent organs.

Statistical analysis was performed using statistical software (Open Epi and Vassar Stats). The sensitivity (Se), specificity (Sp), positive predictive value (PPV), and negative predictive value (NPV) were calculated for both sonography and MRI. The Se and Sp values of sonography and MRI were compared by means of the McNemar test. Se, Sp, PPV and NPV were calculated for each evaluated ultrasound and MRI feature. A p value <0.05 was considered statistically significant.

## Results

42 patients underwent both ultrasound and MRI to explore suspected placenta accreta. Clinical information is shown in Table 1. There were 16 cases of placenta accreta/increta, 10 cases of placenta percreta and 16 cases of non-adherent placenta. Pathologic findings were available for 27 patients. Pathologic examination was not performed in 10 cases because of conservative treatment and in 5 cases because delivery was complete and no postpartum haemorrhage occurred (the five placentas were considered normal).

40 patients had a cesarean delivery and 2 had a vaginal delivery (one medical termination of pregnancy and in one patient vaginal delivery was accepted because MRI wrongfully refuted the diagnosis of placenta accreta suspected at ultrasonography and hemostatic hysterectomy for postpartum hemorrhage had to be performed). 14 women underwent conservative treatment (4 placenta accreta/increta and 10 placenta percreta). 8 had a cesarean hysterectomy and 4 had a hysterectomy later on because of secondary complications.

### Sensitivity and Specificity

Ultrasound successfully diagnosed all 26 cases of placenta accreta. In 10 of 16 women finally ascertained to have a normal placenta, ultrasound wrongfully diagnosed adherent placenta.

MRI successfully diagnosed 20 of the 26 cases of placenta accreta and wrongfully diagnosed 8 of the 16 cases of non-adherent placenta as placenta accreta. For one patient, MRI images could not be interpreted because of fetal movements. We considered this case to be wrongly interpreted negative, because there was a failure to identify placenta accreta and the exam was not useful for the clinical management of the patient.

Diagnostic sensitivity for placenta accreta was 100% for ultrasound and 76.9% for MRI (P = 0.03). Specificities were 37.5% for ultrasound and 50% for MRI (P = 0.6). The diagnosis was correct in 76.2% of cases with ultrasonography and in 66.7% with MRI. The positive predictive value was 72.2% for ultrasound and 71.4% for MRI (see Table 2).

**Table 2:** Sensitivity and specificity of ultrasound and MRI.

	Se	Sp	PPV	NPV	Exact diagnosis
	%, (CI)	%, (CI)	%, (CI)	%, (CI)	%, (CI)
**Ultrasound**	100	37.5	72.2	100	76.2
**n = 42**	(87.1–100)	(18–61)	(56–84)	(61–100)	(61–86)
**MRI**	76.9	50	71.4	57	66.7
**n = 42**	(58–89)	(28–72)	(52.9–84.7)	(32.6–79)	(51–79)
***P*** * ***McNemar test***	0.03	0.6			NS

Se  =  sensitivity, Sp  =  specificity, PPV  =  predictive positive value, NPV  =  negative predictive value.

According to placental insertion, ultrasound correctly diagnosed presence or absence of placenta accreta in 7 cases and MRI in 9 cases of the 11 posterior placenta. So there were no statistical difference between ultrasound and MRI to performe the diagnosis of placenta accreta in case of posterior localization of the placenta (p = 0,26).

### Concordance between Ultrasound and MRI

Ultrasound and MRI were concordant in 28/41 cases (68.3%). In 23 cases, both ultrasound and MRI correctly diagnosed the presence or absence of abnormal adherent placenta (without specifying the depth of the invasion), and in 5 cases both were wrong (5 false-positive diagnoses).

**Figure 5:**
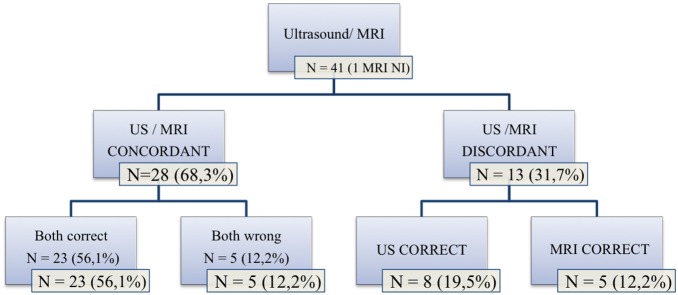
Concordance between ultrasound and MRI.

There was disagreement between ultrasound and MRI in 13 cases, and the sonographic diagnosis was correct in 8 of these cases. Five false-negative results given by MRI were correctly diagnosed by ultrasound. Conversely, in 5/13 cases MRI correctly invalidated a diagnosis of placenta accreta suggested by sonography. These results are shown in figure 5.

When ultrasound and MRI were discordant there were significantly more emergency C-sections and surgeons more ofen attempted placental delivery. However, there was no statistical increase in the rate of cesarean hysterectomy or in the number of blood transfusions. These results are shown in Table 3.

**Table 3:** Consequences of prenatal discordance between ultrasound and MRI.

	Concordance between ultrasound and MRI	Discordance between ultrasound and MRI	*P*
	n = 28	n = 13	
**DELIVERY**			*0.02*
Vaginal delivery	1 (4%)	1 (8%)	*0.53*
Emergency C-section	11 (39%)	10 (77%)	*0.04*
Planned C-section	16 (57%)	2 (15%)	*0.01*
**SURGICAL MANAGEMENT**			*0.056*
Attempted placental delivery	8 (29%)	9 (69%)	*0.01*
Conservative management	14 (50%)	3 (23%)	*NS*
Cesarean hysterectomy	6 (21%)	1 (8%)	*NS*
**TRANSFUSION**			
Number of blood transfusions	10 (36%)	7 (54%)	*NS*
Mean transfused blood volume (in units)	9.5	8	*NS*

### Ultrasound and MRI Features

In order to define the most relevant specific ultrasound and MRI features that may predict placental invasion, ultrasound and MRI images were reassessed by 2 raters with more than 5 years of experience in the evaluation of placentation disorders. All ultrasound images were reassessed (n = 42) and 39 MRI exams were reassessed (1 exam was not interpretable because of fetal movement and for 2 patients MRI images could not be retrieved).

When compared with the appearance of the normal placenta on ultrasound and MRI, 5 features were found to differ statistically significantly between patients with placental invasion and those with normal placentation. These features were loss of the normal retroplacental clear space (P = 0.0004), thinning or disappearance of the myometrium (P = 0.01), increased vascularization at the uterine serosa-bladder wall interface (P = 0.01) and vascularization perpendicular to the uterine wall (P = 0.007) on ultrasonography, and uterine bulging (P = 0.04) on MRI.

On ultrasonography, features which had better sensitivity for the detection of placental invasion were intraplacental lacunae and loss of the normal retroplacental clear space (sensitivity 88%), which respectively had a specificity of 25% and 69%. Increased vascularization in the uterine serosa-bladder wall interface and vascularization perpendicular to the uterine wall had the best PPV (92%). Loss of the normal retroplacental clear space and a pseudo-tumoral appearance of the placenta had a PPV of 82%.

On MRI, thinning or disappearance of the myometrium had the best sensitivity (91%) but a low specificity (13%). Uterine bulging had the best positive predictive value (PPV = 85%), and its combination with the presence of dark intraplacental bands on T2-weighted images improved the predictive value to 90%. A statistically significant difference in the combination of these 2 features was seen between patients with placental invasion and those with normal placentation (P = 0.02). The sensitivity and the predictive values of ultrasound and MRI features are summarized in table 4.

**Table 4:** Sensitivity and predictive values of ultrasound and MRI features.

	Placentaaccreta/percreta (n = 26)	Non-adherent placenta (n = 16)	*P*	Se	Sp	PPV	NPV
ULTRASOUND FEATURES							
Intraplacental lacunae	23	12	*0.39*	88%	25%	66%	57%
Loss of the normal retroplacental clear space	23	5	*0.0004*	88%	69%	82%	79%
Thinning or disappearance of the myometrium	19	5	*0.01*	73%	69%	79%	61%
Thinning or disruption of the hyperechogenic uterine serosa-bladder wall interface	15	6	*0.34*	58%	63%	71%	48%
Increased vascularization at the uterine serosa-bladder wall interface	11	1	*0.01*	42%	94%	92%	50%
Vascularization perpendicular to the uterine wall	12	1	*0.007*	46%	94%	92%	52%
Exophytic uterine masses	11	2	*0.08*	42%	88%	85%	48%
Irregular bladder wall	10	3	*0.3*	38%	81%	77%	45%
Pseudo-tumoral appearance of placenta, uterine bulging	9	2	*0.15*	35%	88%	82%	45%
**MRI FEATURES**	**n = 23**	**n = 16**					
Uterine bulging	11	2	*0.04*	48%	88%	85%	54%
Dark intraplacental bands on T2-weighted images	14	6	*0.2*	61%	63%	70%	53%
Disruption of the interface between placenta and myometrium on T2-weighted images	20	15	*0.63*	87%	6%	57%	25%
Thinning or disappearance of the myometrium	21	14	*1*	91%	13%	60%	50%
Extension of the placenta on T2-weighted images	8	2	*0.15*	35%	88%	80%	48%
Presence of neovessels	6	4	*1*	26%	75%	60%	41%
Dark intraplacental bands and thinning or disappearance of the myometrium	14	6	*0.2*	61%	63%	70%	53%
Dark intraplacental bands and disruption of the interface beetwen placenta and myometrium	12	6	*0.5*	52%	63%	67%	48%
Uterine bulging and dark intraplacental bands	9	1	*0.02*	39%	94%	90%	52%

**Figure 6:**
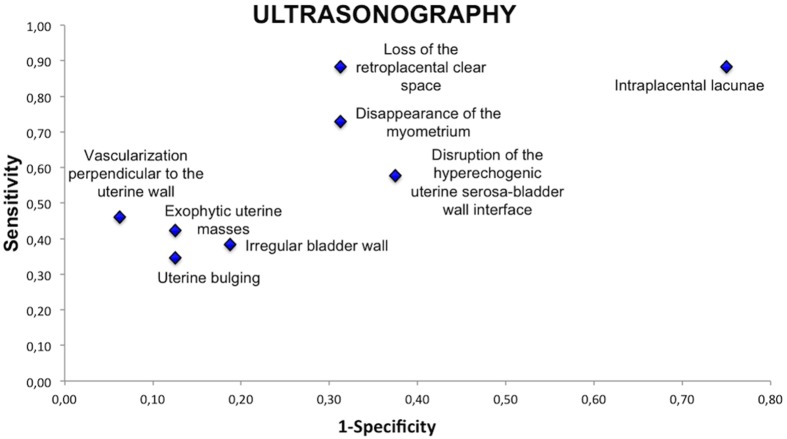
Sensitivity and specificity of ultrasound features.

**Figure 7:**
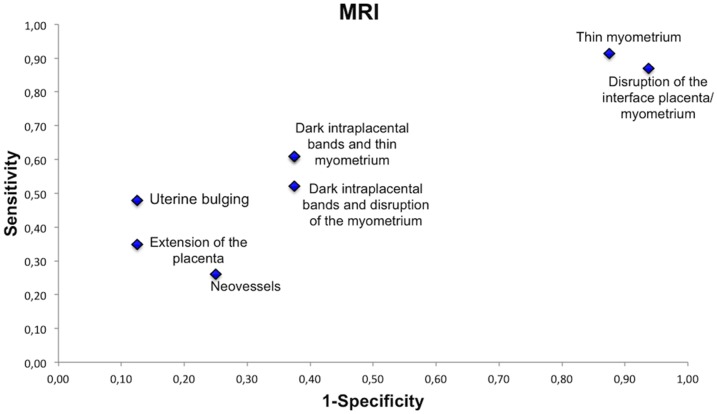
Sensitivity and specificity of MRI features.

In order to visualize the sensitivity and specificity of each feature, we represented these values on receiver operating characteristics curves (Figures 6–7). On ultrasonography, the most relevant features were loss of the normal retroplacental clear space, thinning or disappearance of the myometrium and vascularization perpendicular to the uterine wall. On MRI, the most relevant features were uterine bulging and the presence of dark intraplacental bands associated with thinning or disappearance of the myometrium.

7 MRI scans were done after intravenous injection of gadolinium and for 6 patients MR diffusion-weighted imaging was performed in addition to conventional sequences. There were no statistical differences in the accuracy of MRI for the diagnosis of placenta accreta when using gadolinium injection or MR diffusion-weighted imaging.

## Discussion

Although ultrasound is the mainstay in the imaging of placenta accreta, MRI has been used as an adjunct in diagnosis when the ultrasound results are equivocal and/or clinical suspicion is high. Overall, in our study, the diagnosis of abnormal attachment of the placenta to the myometrium was correct in 76.2% of cases for Doppler ultrasound and in 66.7% of cases for MRI (difference not significant). In the literature, a mixed performance is observed. The sensitivity of Doppler ultrasound ranges from 33 to 100% and its specificity from 50 to 96%, depending on the study[6]–[19]; and the sensitivity of MRI ranges from 38 to 100% and its specificity from 55 to 100% [7]–[13], [15], [16], [18]–[20].

Three recently published meta-analyses have considered the accuracy of ultrasound for the diagnosis of invasive placentation [13], the use of MRI [14] and a comparison of ultrasound and MRI [18]. D’Antonio et al [13], [14] reported a sensitivity of 90.7% for ultrasound and 94.4% for MRI, and a specificity of 96.9% for ultrasound and 84% for MRI. Meng et al [18] showed that ultrasound sensitivity was 83%, and its specificity was 95%, compared with 82% and 88%, respectively, for MRI. These meta-analyses showed good accuracy of ultrasound and MRI in the diagnosis of placental invasion. They comprised several studies and a large number of patients, but also included studies that were clinically and methodologically varied, and in which ultrasound and MRI were not applied to the same population. This may represent an unavoidable source of bias. The results are only applicable to women with placenta previa and a history of a cesarean delivery or uterine surgery.

These 3 meta-analyses reported that ultrasound and MRI are equally accurate in diagnosing the presence of invasive placentation. We found a statistical difference in sensitivity between MRI and ultrasound, but no difference in specificity or in the percentage of correct diagnoses. This statistical difference might have arisen because only when the placenta was suspected to be adherent on ultrasound was the patient referred for MRI, thus increasing the specificity of MRI and decreasing its sensitivity.

Compared with the literature, we found a better sensitivity but a lower specificity of ultrasound for the diagnosis of placenta accreta, perhaps because, as in Comstock et al. [21], we considered the placenta to be accreta as soon as one feature was present. This increases the number of false positives and reduces the specificity of the test [13], [22].

Several authors found a better performance of MRI compared to ultrasound to diagnose placenta accreta when placenta have a posterior insertion [12], [23]–[25]. Our study did not found difference between these two imaging techniques in this condition.

Many authors consider the presence of intraplacental lacunae to be the best ultrasonography feature [7], [9], [11]–[13], [17], [21], [26]–[28]. In our study, we also found a good sensitivity for this feature, but its specificity and PPV were low. In the presence of this feature we must pay attention to abnormal placentation, especially in the case of low-lying anterior insertion of the placenta and history of cesarean section, but it is not pathognomonic for placenta accreta. Its combination with other features increases its PPV. Lacunae may be present even in women with placenta previa without myometrial invasion [22], [29], but their presence increases the risk of hemorrhage at delivery [30].

**Figure 8:**
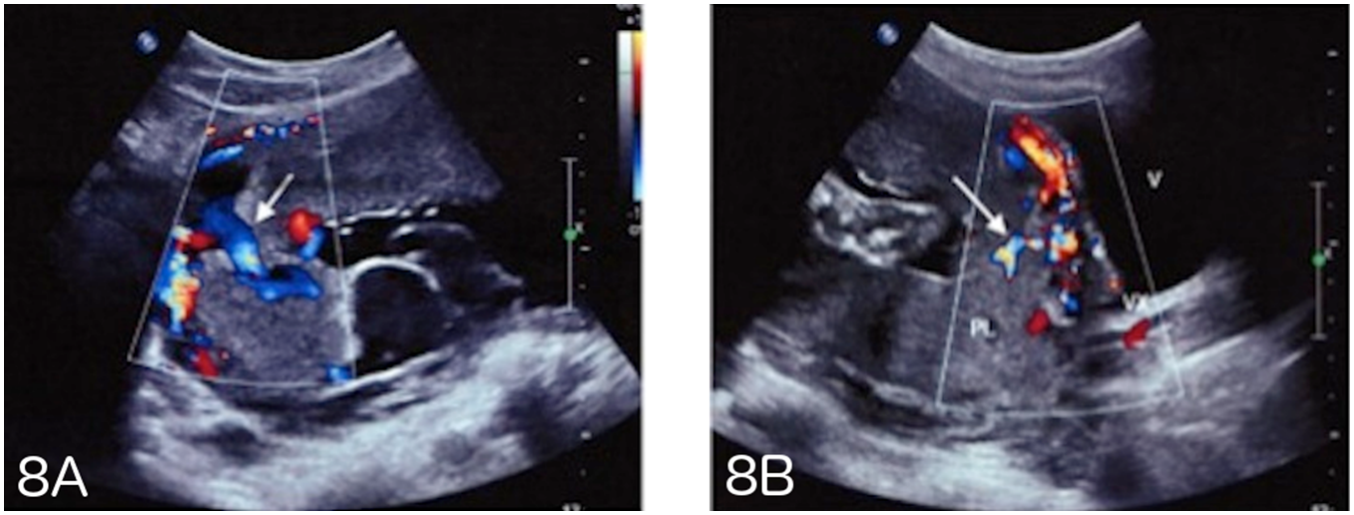
A–B - Intraplacental vascularization perpendicular to the myometrium and hypervascularization on ultrasound.

Vascularization perpendicular to the myometrium, a feature used by our teams (Figure 8), had a positive predictive value of 92% and appears to be one of the most discriminating characteristics for the diagnosis of placenta accreta. It reflects the loss of the normal architecture of the vessels of the placenta with intra-placental hypervascularization and chaotic connections. Other authors have also reported that abnormal vascularization seen by color Doppler ultrasound has the best combination of sensitivity and specificity and that its localization at the uterus-bladder interface has the best specificity in the prediction of invasive placentation [13], [15], [22].

The retroplacental hypoechoic clear zone represents the thickness of the decidua basalis. On ultrasonography, its disappearance literally reflects the histological observation in the case of placenta accreta. The sensitivity and PPV observed for this feature in our study are higher than those found in the literature [13], [21]. It is also the feature with the best NPV in our patient group. Cali et al. found the same results [22]. They underlined that as it had a good NPV, if the echolucent area between the placenta and the uterus is preserved, morbidly adherent placenta is unlikely to occur. It is, however, difficult to see and ideally requires a high-frequency probe oriented perpendicularly to the myometrium/placenta interface and an experienced operator. It is also interesting to measure the distance over which this zone is absent since it can be used to assess the area of abnormal adherent placenta.

As in the literature [14], [20], [22], [23], [31]–[34], we found the best PPV (90%) of MRI when dark intraplacental bands were associated with disappearance of the myometrium and uterine bulging. Lim et al. also showed that the volumes of dark intraplacental bands on T2-weighted images were significantly different in the patients with abnormal placentation and without placenta accreta (p = 0.047), and that band volumes were differed significantly between patients with accreta, increta, and percreta (p<0.0005)[12].

We have evaluated the performance of two imaging techniques used in the prenatal diagnosis of placenta accreta in the same patient population. The accreta or percreta characteristic of the placenta was based on pathological examination, which is more reliable than intraoperative surgical findings. It also specifies the diagnostic value of each feature for both imaging techniques. However, this study is retrospective, implying that the evaluation of ultrasound and MRI imaging features was done retrospectively, but without knowing the final diagnosis. With MRI this did not change the result, but we are aware that for Doppler ultrasound the absence of a dynamic study is a limitation.

We did not show an increased accuracy of MRI when using gadolinium or MR diffusion-weighted imaging. Warshak et al. [9] used gadolinium because they thought that it improved the specificity of the technique as it delineates the outer placental surface proximal to the myometrium more clearly. The European Medicines Agency recommends that contrast MRI be used with caution in pregnant women, and only if the benefits outweigh the risks [35].

Ultrasonography remains the most sensitive and commonly used imaging modality for the diagnosis of placenta accreta, because it is accurate, inexpensive, non-invasive and time-saving. MRI appears to be complementary to ultrasonography, especially when there are few ultrasound signs. In such cases, it is important to assess the value of each feature according to its PPV, but also according to the NPV of absent characteristics. In these situations MRI appears to be helpful because it can reveal signs not visible by ultrasound (dark intraplacental bands, for example) which can be used to confirm or refute the diagnosis of placenta accreta. On the other hand, if there is a strong suspicion of placenta accreta or percreta at Doppler ultrasound, with several signs present with good PPV, the result of the MRI exam should not alter the obstetric management [36]. Because of the possible burden for the patient in the case of placenta accreta, she should be referred to an appropriate institution for perpartum management and the placenta should be considered as accreta when organizing the course of delivery.
